# Replacing Quantum Chemistry With Machine-Learned Interatomic Potentials: Revolution or Evolution?

**DOI:** 10.1021/acscentsci.6c00615

**Published:** 2026-06-23

**Authors:** Andrew J. Medford, David S. Sholl

**Affiliations:** † School of Chemical & Biomolecular Engineering, 1372 Georgia Institute of Technology, Atlanta, Georgia 30332, United States; ‡ Department of Chemical & Biomolecular Engineering, 3990 Rice University, Houston Texas 77005, United States

## Abstract

A long-standing goal in computational chemistry and materials science has been the development of general-purpose interatomic force fields that define the energy and forces associated with arbitrary sets of atoms. This task can be accomplished with density functional theory (DFT) and other levels of computational quantum chemistry, but the computational cost of these methods strongly constrains the physical problems that can be explored. Rapid advances in machine-learned interatomic potentials (MLIPs) mean that calculations at DFT levels of accuracy will soon be accelerated by factors of up to a million, a situation that will dramatically change the landscape of computational chemistry. In this Outlook, we examine the implications and limitations of MLIPs and describe a research agenda for taking full advantage of these remarkable tools.

## Graphical abstract

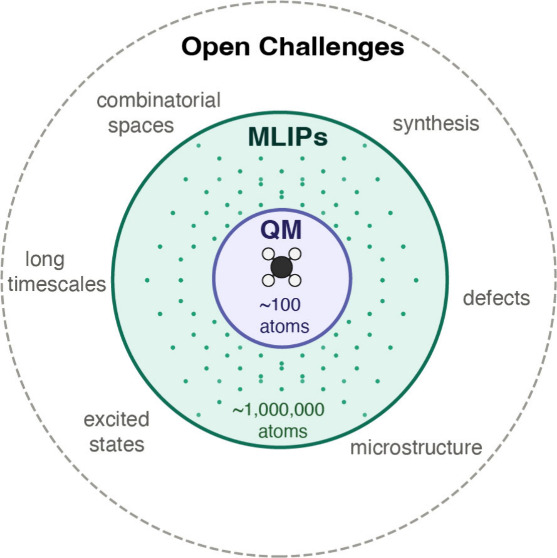

The Schrödinger equation defines how quantum mechanics leads to the properties of all the molecules and materials that surround us. Computational quantum chemistry, the approximate numerical solution of the Schrödinger equation, plays a vital role in modern chemistry and materials science. John Pople and Walter Kohn were awarded the Nobel Prize in Chemistry in 1998 for developing methods that founded this field. Tens of thousands of scientific papers using quantum chemistry are published each year. The successes of this field have been enormous, but quantum chemistry calculations remain computationally demanding, often limiting the ability of researchers to address complex experimentally relevant situations.

A central task in quantum chemistry is to calculate the energy of a collection of atoms and the forces on each atom due to all other atoms (see Figure 1). This information, known as the interatomic potential, can be exploited to determine a wide range of microscopic and macroscopic physical properties. Ideally, calculation of interatomic potentials for the full spectrum of elements from the periodic table and the complete set of chemical bonds and other interactions between atoms would be possible without the computational challenge of solving the Schrödinger equation. Many efforts to achieve this ideal have been made,^1^ yet no approach that can rival computational quantum chemistry as a “universal materials calculator” has emerged.

**Figure 1:**
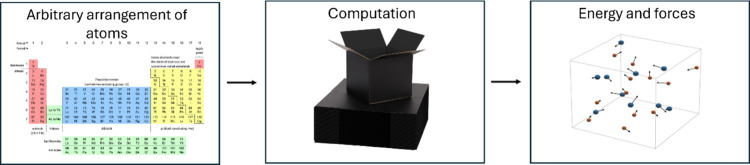
Interatomic potentials define the energy and forces associated with collections of atoms. General purpose interatomic potentials can be applied to any set of atoms (image credits: Wikimedia Commons under Creative Commons license and ucanpack.com).

Quantum chemistry is rapidly going through a revolution that will allow interatomic potentials to be computed with vast increases in speed without significant reductions in accuracy. The central idea driving this change is to develop interatomic potentials by applying machine learning methods to very large collections of quantum chemistry calculations. The resulting methods, Machine-Learned Interatomic Potentials (MLIPs), may represent a pivotal inflection point in a scientific pursuit with broad implications for discovery of materials and chemicals. Although many questions in this rapidly developing field remain open, it is reasonable to anticipate that MLIPs will soon effectively replace many quantum chemistry calculations.^2–5^

Although many questions in this rapidly developing field remain open, it is reasonable to anticipate that MLIPs will soon effectively replace many quantum chemistry calculations.

Although there is wide variability, quantum chemistry can today routinely be applied to problems containing tens or hundreds of atoms. We expect that MLIPs will reduce computation times relative to quantum chemistry by factors as large as 10^6^. This outcome will completely redefine what a readily accessible calculation looks like and further democratize the availability of these calculations to nonspecialists. At the same time, rapid changes in scientific capabilities create significant challenges to a scientific community’s collective imagination since any human endeavor is beset by inertia and expectations of incremental change. Below, we discuss what will (and will not) soon be possible using MLIPs and the key challenges researchers will face in adapting these tools to solve demanding real-world problems.

## MLIPs Will Not Change the Research Landscape for Problems Currently Tackled Using Classical Potentials

MLIPs are not a panacea that will supersede all other methods for modeling materials on atomic length scales. Interatomic potentials are already widely used to study systems involving 10^4^–10^6^ atoms by relying on “classical” empirical potentials instead of quantum chemistry. We will refer to these methods as classical molecular simulations for simplicity, and they are applicable to many interesting fundamental and applied phenomena.^6^ MLIPs are multiple orders of magnitude more computationally intensive than typical classical potentials and this situation is unlikely to change in the future.^7,8^ For this reason, MLIPs will not revolutionize problems that can currently already be described with large-scale classical molecular simulations. It is also important to note that there are many phenomena that occur on length and time scales far larger than those accessible in classical molecular simulations, and MLIPs will not have an impact on modeling these phenomena. Taking advantage of MLIPs to model collections of 10^4^–10^6^ atoms instead of the 10^1^–10^3^ atoms accessible with quantum chemistry today requires changes in the mindset of researchers, and the decades of experience available in the molecular simulation community will be critical to enabling these changes.

## MLIPs Provide Full Access to Chemical Reactions, and This Brings Challenges in Sampling Phase Space

A vital distinction between classical molecular simulations with interatomic potentials suitable for species from large portions of the periodic table and quantum chemistry is that only the latter can readily describe chemical reactions, that is, the formation or breaking of chemical bonds. The possibility of using MLIPs to explore the full range of chemistry possible with complex collections of atoms and molecules will have an enormous impact. A key issue in simulating any complex array of atoms is how to sufficiently sample the enormous number of possible arrangements of the atoms, that is, the phase space. In molecular simulations that do not involve chemical reactions, it is often possible to sample phase space using molecular dynamics (MD), which relies on thermal vibrations to overcome modest energy barriers that separate nearby energy minima in phase space. There are many examples, however, where these energy barriers are larger than thermal energies, and a host of enhanced sampling methods have been developed for these situations.^6^ This challenge is greatly exacerbated in systems involving chemical reactions, which typically have larger energy barriers and much more spatially localized energy minima. To reap the full benefits of MLIPs, it is important to apply and improve enhanced sampling techniques for reactive systems. The challenge of adequately sampling phase space is also central to developing training data for MLIPs. MLIPs relying on training data that focused exclusively or primarily on equilibrium states (e.g., energy-minimized structures) cannot be expected to make accurate predictions about nonequilibrium states such as transition states. Addressing the challenge of creating training data with sufficient complexity is likely to be a long-term challenge for these methods.

## Speeding up MLIPs Will Be an Important Area for Future Developments

As general-purpose MLIPs become available, approaches that can further reduce their computational cost will become an area of substantial interest.^9^ It seems likely that efficiency increases of orders of magnitude will be possible by combining model distillation techniques relevant to all large ML models along with insights that leverage the physical characteristics of simulating atoms and molecules. For example, it may be advantageous to sample phase space using low-cost/moderate-accuracy models that are updated at an appropriate frequency by a high-cost/high-accuracy model. Similarly, it may be strategic to use high-cost general-purpose models to create low-cost distilled models that are appropriate for more specific chemistries. These trade-offs among speed, accuracy, and generality for MLIPs are currently not well explored.

As general purpose MLIPs become available, approaches that can further reduce their computational cost will become an area of substantial interest.

## Chemical Search Spaces Are Vast and Will Often Defy Brute Force Search Strategies Relying on MLIPs

A central goal in many areas of chemistry or materials science is to find the best, or at least a high performing, molecule or compound for some desired property. Small molecule drug discovery is one example of this situation. The collection of possible molecules or materials that could be considered is described as the chemical space. A central attribute of chemical spaces is that they contain vast numbers of different compounds. Data sets with more than 10^11^ organic molecules with modest molecular weights are available.^10^ For octahedral transition metal complexes with a single metal atom and a small number of coordinating ligands, chemical spaces with 10^21^ cases exist.^11^ Although MLIPs will be enormously faster than quantum chemistry, they will not make it possible to enumerate chemical spaces of this kind by brute force computation. The possibility of using MLIPs to generate accurate predictions for large numbers of materials or compounds, however, opens the door to combining this data with other search strategies. For example, MLIPs can be combined with techniques such as descriptor-based cheminformatics or generative models to rapidly and strategically expand searches of chemical space.^12–14^ We anticipate that powerful advances will be made in coming years that creatively combine approaches in this way.

## Predicting Chemical and Material Structures Is (Much) Easier than Actually Making Them

Predicting the properties of a new molecule or material computationally is just one possible way to identify new materials. The true test of whether a material can be used is whether it can be produced experimentally.^15^ As with all materials modeling techniques, users of MLIPs need to remain acutely aware of the many real-world complications that can compromise the properties of a material. We strongly suggest that modelers actively articulate a list of complications that could limit connections between computational predictions and real materials; work that does not do this is likely to have a limited impact.

Creatively spanning the gulfs that can exist between computational predictions and experimental reality is an area of huge opportunity. Because MLIPs will allow large changes in the complexity of problems that can be addressed, they will allow the computational research community to reconsider the effects of defects, disorder, and synthesis procedures that previously have been ignored because they were computationally inaccessible. This challenge is too important to be left to the modeling community alone, and we hope that experimenters will make extravagant demands on their colleagues who aim to use MLIPs in nonincremental ways.

Creatively spanning the gulfs that can exist between computational predictions and experimental reality is an area of huge opportunity.

## MLIPs Are Only As Good As Their Training Data, So Verification of Key Results Will Remain Vital

The aim of MLIPs as we have described them is to provide quantum chemistry results with drastically reduced computational effort. This description elides the complexity of quantum chemistry methods, which are better described as a diverse array of related methods with varying levels of precision with respect to the goal of numerically solving the Schrödinger equation. High quality MLIPs will be based on large data sets of quantum chemistry calculations, but developments and controversies regarding selection and comparison of the specific quantum chemistry methods have persisted for many years. To give just one of many possible examples, DFT calculations are typically not effective in describing weak van der Waals interactions, so MLIPs trained exclusively on data of this type would also not capture these interactions. Other related issues have recently been reviewed in detail by Li et al.^16^ These challenges will persist and be magnified as the curated data sets used to train and validate MLIPs expand. Moreover, even the best MLIPs will not generate highly precise predictions at the relevant level of quantum chemistry for all possible arrangements of atoms, and robust uncertainty quantification for MLIPs has proven difficult.^17,18^ The materials and chemistry research community should expect users of MLIPs to test the accuracy of the most interesting predictions made with MLIPs by direct comparison with quantum chemistry data. Although this idea seems simple, it will increasingly become more subtle as MLIPs are applied to large and complex cases that, by design, are computationally inaccessible with quantum chemistry calculations.

## Chemistry and Material Properties Involve More than Atom Positions and Forces

We defined the goal of MLIPs as providing interatomic potentials, that is, the energy and forces, defined by an arbitrary collection of atoms. There are of course many interesting properties of molecules and materials that cannot be determined from energy and forces alone. The classification of materials as insulators, semiconductors, or metals relies on the band gap, a property of the electronic wave function. Understanding what makes some solids high *T*_c_ superconductors is a topic of active debate and certainly cannot be determined from energies and forces. A more subtle issue is that a given configuration of atoms can define multiple energies by considering excited states, spin states and so on. As a rule of thumb, if meaningful quantum chemistry calculations for a property of interest are challenging in small systems for experts with today’s methods, then predicting these properties will not be “solved” by switching to MILPs.

## Conclusion

MLIPs are revolutionizing what is possible in computational chemistry and materials science by expanding the size and length scales that are accessible for atomistic simulations that can include chemical reactivity and apply to (almost) all elements in the periodic table. This capability represents a dramatic step forward in the application of computation for chemical and materials discovery, although it will not directly solve the fundamental challenges related to vast search spaces, complexities that distinguish between computational models and real systems, or quantum-mechanical phenomena. Experts who have been trained in computational quantum chemistry have become used to incremental improvements in available methods, but this situation could create an inadvertent cultural roadblock to being able to take full advantage of the stunning acceleration that MLIPs will allow. Success in this area will require individual and collective intellectual bravery, creative problem formulation, and a willingness to learn from other disciplines.

The incredible successes of AlphaFold for predicting protein structure, whose developers shared the 2024 Nobel Prize in Chemistry, offer a useful analogy for the possibilities created by MLIPs. AlphaFold used machine-learning approaches trained on enormous collections of known protein structures obtained from thousands of person-years of meticulous experiments, converting this knowledge into a tool that researchers can use to rapidly predict folded conformations from protein sequences. Although this capability was transformational, it is only half of the story. The other half of the 2024 Nobel Prize went to David Baker, who pioneered the development of algorithms and experimental techniques to both predict and realize novel proteins in the laboratory. Protein design has flourished even after the development of AlphaFold and will continue to discover new horizons. As researchers in computational materials and chemical discovery use MLIPs to explore larger chemical spaces and push to larger time and length scales, new concepts and insights will undoubtedly emerge. Nevertheless, human insight and innovation will continue to be vital as the field adapts to these powerful new tools.
